# Associations of Frailty Status with Low-Mileage Driving and Driving Cessation in a Cohort of Older Drivers

**DOI:** 10.3390/geriatrics5010019

**Published:** 2020-03-19

**Authors:** Christopher L. Crowe, Sneha Kannoth, Howard Andrews, David Strogatz, Guohua Li, Carolyn DiGuiseppi, Linda Hill, David W. Eby, Lisa J. Molnar, Thelma J. Mielenz

**Affiliations:** 1 Department of Epidemiology, Mailman School of Public Health, Columbia University, New York, NY 10032, USA; clc2229@cumc.columbia.edu (C.L.C.); sk4690@cumc.columbia.edu (S.K.); gl2240@cumc.columbia.edu (G.L.); 2 Department of Biostatistics, Mailman School of Public Health, Columbia University, New York, NY 10032, USA; howard.andrews@nyspi.columbia.edu; 3 Bassett Research Institute, Center for Rural Community Health, Cooperstown, NY 13326, USA; david.strogatz@bassett.org; 4 Department of Epidemiology, Colorado School of Public Health, Aurora, CO 80045, USA; Carolyn.DiGuiseppi@cuanschutz.edu; 5 Department of Family and Preventive Medicine, University of California San Diego, San Diego, CA 92161, USA; llhill@ucsd.edu; 6 Transportation Research Institute, University of Michigan, Ann Arbor, MI 48109, USA

**Keywords:** frailty, driving mobility, low-mileage, driving cessation, independence

## Abstract

The US older adult population is projected to considerably increase in the future, and continued driving mobility is important for health aspects in populations with fewer transportation alternatives. This study evaluated whether frailty is associated with low-mileage driving (<1865 miles per year) and driving cessation among older adults. Baseline demographics and health data were collected for 2990 older drivers via in-person assessments and questionnaires, with 2964 reporting baseline frailty data. Multivariable log-binomial regression models were used to evaluate the association between baseline frailty status and low-mileage driving. Multivariable Cox proportional hazards regression were used to evaluate the association between baseline frailty status and driving cessation. For every unit increase in frailty, the estimated adjusted risk of driving fewer than 1865 miles/year increased by 138% (adjusted risk ratio: 2.38, 95% CI: 1.63–3.46). Relative to older drivers who were not frail, the adjusted hazard ratios of driving cessation were 4.15 (95% CI: 1.89–9.10) for those classified as prefrail and 6.08 (95% CI: 1.36–27.26) for those classified as frail. Frailty is positively associated with low-mileage driving status and driving cessation in a dose-response fashion. Public health interventions that reduce frailty, such as physical activity, may help older drivers maintain safe and independent mobility.

## 1. Introduction

By 2050, the number of adults aged 65 years and older (“older adults”) in the U.S. is expected to double to approximately 90 million. As more Americans continue to drive during late adulthood, it is predicted that a quarter of all licensed drivers in 2050 will be older adults [1]. Given that continued mobility is essential to maintaining one’s ability to remain integrated in society and complete the tasks required for daily living, low-mileage driver status and driving cessation will present a major threat to physical, social, and mental health for many of these older adults [2,3,4,5], especially those who lack access to alternative transportation. Additionally, low-mileage driver status is known to be a risk factor for higher crash rates per mile driven [6]. As motor vehicle crashes represent the second most common cause of injury among older adults [1], the association with higher crash rates further highlights the importance of identifying risk factors for reduced driving exposure. While there may be a number of factors that influence one’s driving exposure, one particularly interesting hypothesis is that an increase in older adult’s frailty status predicts decreased driving exposure [6,7]. It is important to assess how potentially modifiable frailty may specifically predispose older adults to low-mileage driving status and eventual driving cessation, so that interventions may be appropriately designed to target those who need them most.

Frailty is a clinical syndrome that develops following a decline in function and resilience across numerous physiological systems [8,9,10,11]. One commonly used measure of frailty is the frailty phenotype created and validated by Fried and colleagues, which provides a standardized measure based on the assessment of five criteria: unintentional weight loss, weakness, exhaustion, slowness, and low physical activity [9]. The presence of frailty among older adults is known to be associated with an increased risk of adverse health outcomes, such as falls, incident disability, hospitalization, and crash-related injury or death [9,10,11,12]. However, there is a distinct lack of information about how frailty phenotype impacts driving exposure.

This study aimed to empirically establish frailty as a risk factor for adverse driving outcomes by examining how higher levels of frailty phenotype may predict (1) annual mileage and (2) driving cessation. It was hypothesized that higher levels of the frailty phenotype would be associated with low-mileage driver status and a higher rate of driving cessation, after adjusting for demographics and other characteristics known to be associated with driving exposure.

## 2. Materials and Methods

### 2.1. Study Population

The data used in this study are from the American Automobile Association (AAA) Longitudinal Research on Aging Drivers (LongROAD) study, a multisite prospective cohort study. The study population and data collection methods are summarized here and described in full detail elsewhere [13]. A total of 2990 participants were recruited from five sites: Ann Arbor, MI; Baltimore, MD; Cooperstown, NY; Denver, CO; and San Diego, CA. To be included in the study, participants were required to be between 65 and 79 years of age, have a valid license, drive at least once a week, have no plans to move away from the study site that would prevent follow-up, spend at least 80% of the time driving a single vehicle of model year 1996 or newer, be fluent in English, and be free of any significant cognitive impairment. Eligible participants who provided consent were assessed at baseline via in-person visits at the study site. For the first annual follow-up visit, data were collected via abbreviated telephone interviews. Data from performance-based assessments were recorded during in-person visits. Up to almost three years of objective driving behavior data were collected via in-vehicle devices. The research was approved and monitored by the Institutional Review Board of the Columbia University Medical Center [IRB-AAAN9950].

### 2.2. Predictors

#### Frailty Status

Frailty was measured using the frailty phenotype [9,11,14,15,16,17]. In accordance with these guidelines, frailty status was evaluated by assessing five criteria on a pass/fail basis: shrinking (unintentional loss of ≥10 pounds in the past year or being underweight according to a BMI of ≤18.5 kg/m^2^), weakness (grip strength in the lowest 20% of the population, adjusted for gender and BMI), exhaustion (self-report of having poor endurance and energy), slowness (slowest 20% of the population based on time to walk 15 feet, adjusted for gender and standing height), and low physical activity (not having recently walked for exercise or engaged in vigorous physical activity). One point was given for each criterion the participant exhibited and scores were categorized as not frail (0), prefrail (1–2), or frail (3–5) [9,10,14]. Of the 2990 participants in the AAA LongROAD cohort, 2964 had baseline data for frailty status.

### 2.3. Outcomes

#### 2.3.1. Low-Mileage Driver Status

Annual mileage was measured using DataLogger (Danlaw, Inc., Novi, MI, USA), an in-vehicle device installed in the participant’s primary vehicle after receiving informed consent. This device is designed to record Global Position System (GPS) and accelerometer data that are processed to obtain objective driving measures, such as distance traveled, vehicle speed, location, and trip start/end time. These data were transmitted via a cellular connection to a secure server at the University of Michigan Transportation Research Institute for data management at the end of each trip. Since a participant’s primary vehicle may be shared among multiple drivers, the participant and other potential drivers were instructed to carry Bluetooth low energy cards that broadcasted unique identification (ID) codes. The DataLogger was equipped with a Bluetooth receiver that recorded these IDs and the associated signal strengths, to determine who was driving the vehicle during each trip. Data corresponding to drivers other than the participant were removed, and the retained data produced 31 monthly summary variables for each participant at the end of each month of driving [13]. Using these data, a prorated variable was derived to measure each participant’s annual driving mileage. This variable was calculated by dividing a participant’s total number of miles driven by the number of months of recording and multiplying by 12. Those with at least 12 months of driving data were included in the analyses for this study.

The variable of miles per year was dichotomized two ways. First, low-mileage drivers were defined as those who drove fewer than 3000 miles per year in accordance with the values used by Antin et al. (2017) and other research groups [6,18]. Second, low-mileage drivers were defined as those who drove fewer than 1865 miles (approximately 3000 kilometers) per year based on the threshold used in other studies [18,19]. Of the 2990 participants in the AAA LongROAD cohort, 2807 had at least 12 months of driving behavior data, which amounted to 61,528 person-months of data for the analyses.

#### 2.3.2. Driving Cessation

Participants were asked about driving status at each follow-up and were instructed to notify the research team at their study site if they transitioned from driver to nondriver status at any timepoint in the study. Furthermore, DataLogger activity was monitored for all participants, and participants who did not have any driving activity for at least 30 days were contacted by the site research team in order to identify a permanent change in driving status. The date of driving cessation was recorded accordingly. Cases of driving cessation were defined as either (1) those who voluntarily stopped driving with no intention of becoming an active driver again or (2) those who involuntarily stopped driving with no expectation of becoming an active driver again. Of the 2879 participants who were included in the final analyses, 27 participants reported a driving cessation event.

### 2.4. Covariates

Important variables to be considered as potential confounders were determined a priori and included age, gender, education level, marital status, self-reported vision, depression, performance-based cognitive health, and driving importance. Depression, education level, marital status, and self-reported vision were recorded at both baseline and the first-year follow-up visit; all other covariates were assessed at baseline.

Depression was scored using the Patient-Reported Outcomes Measurement Information System (PROMIS) instruments, with higher scores indicative of more severe depression [20]. Cognitive health was assessed by performance on immediate and delayed word recall tasks, which are designed to test episodic and working memory, with higher scores indicative of better cognitive health [21,22]. To measure driving importance, participants were asked “How important is it that you continue driving?”. Responses ranged from 1 (not at all) to 7 (completely), and were divided into three categories: 1 to 5, 6, and 7 [22]. Age at baseline was categorized as 65–69, 70–74, and 75–79; gender was treated as a binary variable including male and female; highest level of education attained was categorized as high school or less, some college, bachelor’s degree, and advanced degree; marital status was categorized as married/living with partner, separated/divorced/never married, and widowed; self-reported vision was categorized as poor to good, very good, and excellent; depression was dichotomized at a T score value of 55; and cognitive health was dichotomized at the median [22,23].

### 2.5. Statistical Analyses

Descriptive statistics were used to examine the distribution of the exposures, outcome, and covariates in the AAA LongROAD cohort. Bivariate analyses were conducted using Pearson’s chi-squared tests of independence.

Log-binomial regression models were used to model the relationship between frailty status and low-mileage driver status. Two models were created for the exposure of frailty status (one model for the outcome of ≤3000 miles per year and one model for the outcome of ≤1865 miles per year for each exposure of interest). The final adjusted model controlled for potential confounding. Both the crude and the adjusted models used robust standard error estimates to account for potential clustering by site. Frailty status was included in the model as an ordinal variable; all other categorical variables were included as nominal variables due to nonlinearity of beta estimates.

Cox proportional hazards models were used to model the relationship between frailty status and time to driving cessation. The final adjusted model controlled for potential confounding. Both the crude and the adjusted models used robust standard error estimates to account for potential clustering by site. All categorical variables except for frailty status, education, and vision were included in the model as ordinal variables; frailty status, education, and vision were included as nominal variables due to nonlinearity of beta estimates. Depression, marital status, and vision were recorded at both the baseline visit and the first follow-up visit one year later, and were treated as time-dependent covariates.

Given that gender, vision, and cognitive health are known to be associated with driving outcomes, it was decided a priori that these three variables would be included in the final models [24,25]. The remaining covariates were assessed for evidence of confounding using the change-in-estimate method [26]. A cross-product term was used to represent statistical interaction between gender and frailty status on the multiplicative scale in assessing the relationship between frailty and driving cessation. A likelihood ratio test was used to determine whether or not this interaction term should be included in the final model.

All analyses were conducted using Stata version 15 [27]. The level of significance was set at 0.05 for all statistical tests.

## 3. Results

### 3.1. Low-Mileage Driver Status Outcome

More than half (55.87%) of the 2964 drivers were classified as prefrail, while 86 (2.90%) were classified as frail (Table 1). Of the 2807 drivers who had at least 12 months of driving data, either 179 (6.38%) or 50 (1.78%) qualified as low-mileage drivers, using cut-offs of ≤3000 and ≤1865 miles per year, respectively. Age and depression status were significantly associated with frailty status (*p* = 0.013 for age; *p* < 0.001 for depression).

#### 3.1.1. ≤3000 Miles per Year

Age, education, and marital status were significantly associated with being a low-mileage driver using a cut-off of ≤3000 miles per year (data not shown). Likelihood ratio tests comparing the saturated models to the reduced models with no interaction terms found that there were no significant interaction terms (*p* = 0.3802 for frailty model).

After adjusting for the confounders and clustering by site, there was a 36% increase in the risk of low-mileage driver status, using a cut-off of 3000 miles per year (RR = 1.36; 95% CI: 1.11–1.65) for every unit increase in frailty (Table 2).

#### 3.1.2. ≤1865 Miles per Year

Age and marital status were significantly associated with being a low-mileage driver using a cut-off of ≤1865 miles per year. Likelihood ratio tests comparing the saturated models to the reduced models with no interaction terms found that there were no significant interaction terms (*p* = 0.7841 for frailty model).

After adjusting for the appropriate confounders and clustering by site, there was a 138% increase in the risk of low-mileage driver status, using a cut-off of 1865 miles per year (RR = 2.38; 95% CI: 1.63–3.46) for every unit increase in frailty (Table 2).

### 3.2. Driving Cessation Outcome

Based on bivariate analyses, age, depression, and driving importance were significantly associated with frailty status (age: *p* = 0.014, depression: *p* < 0.001, driving importance: *p* = 0.017). Depression, driving importance, and frailty status were significantly associated with time to driving cessation (depression: *p* = 0.0125, driving importance: *p* = 0.0001, frailty status: *p* = 0.0009). The associations between time to driving cessation and age and marital status were not significant (age: *p* = 0.0847, marital status: *p* = 0.0758).

**Figure 1:**
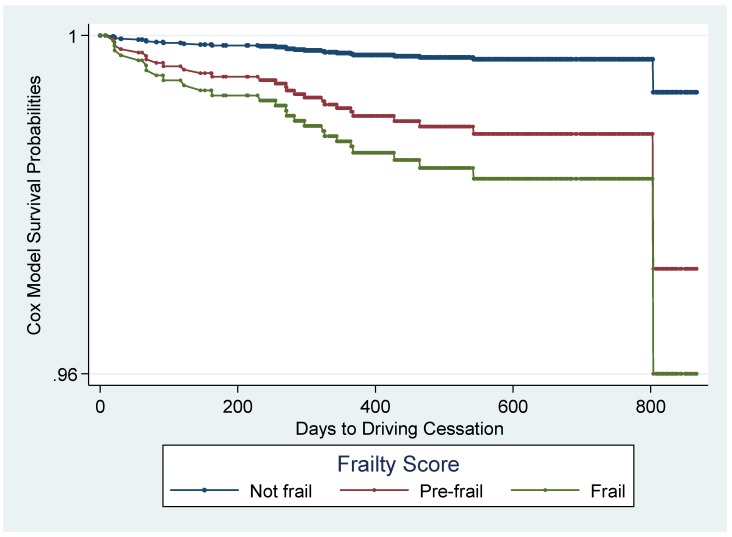
Predicted survival curves by frailty status, adjusted for gender, vision, cognition, and driving importance (N = 2879).

The global test of the Schoenfeld residuals for all covariates was nonsignificant (*p* = 0.4894), thus indicating that the proportional hazards assumption was not violated. Based on the crude association between frailty status and time to driving cessation, the hazard ratio comparing prefrail to not frail was estimated to be 4.04 (95% CI: 1.72–9.46) and the hazard ratio comparing frail to not frail was estimated to be 8.25 (95% CI: 2.08–32.75). After adjusting for gender, self-reported vision, cognitive health, and driving importance, the hazard ratios for both levels of frailty status were statistically significant: 4.15 (95% CI: 1.89-9.10) comparing prefrail to not frail and 6.08 (95% CI: 1.36–27.26) comparing frail to not frail (Table 3). These associations are apparent in the adjusted predicted survival curves estimated from the Cox model, which indicates an accelerated decline in survival for those who are prefrail and an even more rapid decline for those who are frail (Figure 1). The likelihood ratio test indicated that the interaction term between frailty status and gender was nonsignificant (*p* = 0.075), so there was no evidence of interaction on the multiplicative scale, and this term was not included in the final model. 

## 4. Discussion

This study aimed to establish frailty as a risk factor for adverse driving outcomes, specifically low-mileage driver status and driving cessation. The findings demonstrated that every unit increase in frailty corresponded with a 36% increased risk of low-mileage driver status, using a cut-off of 3000 miles per year, and a 138% increased risk of low-mileage driver status, using a cut-off of 1865 miles per year. Similarly, the findings indicated that those participants who were classified as prefrail had 4.15 times the hazard of driving cessation of those who were classified as not frail, and participants who were classified as frail had 6.08 times the hazard of driving cessation of those who were classified as not frail, adjusting for gender, self-reported vision, cognitive health, driving importance, and clustering by site.

Based on the associations found in this study, it appears that the Fried’s frailty phenotype may be useful in identifying older adults who are at an increased risk of low-mileage driver status and driving cessation. This finding agrees with the work of Bond et al. (2017), which found that drivers with frailty have higher rates of becoming nondrivers [28], so it is intuitive that among those who still drive, those with higher levels of frailty are likely to drive less.

Given previous research, the finding that those with higher levels of the frailty phenotype are more likely to be classified as low-mileage drivers implies that measures of the frailty phenotype may also be used to identify older adults who are more likely to be involved in car crashes. This implication is supported by previous research on the AAA LongROAD cohort by Man et al. (2020), which found that being classified as nonfrail was protective against having a self-reported crash rate of one or more crashes in the previous year [29]. If true, this means interventions that aim to reduce the prevalence of frailty may be successful in helping older adults increase their driving exposure [30,31], decrease their overall crash rate, and maintain their driving independence by delaying driving cessation.

It is important to note the limitations of the current study, in that few (<3%) participants were classified as frail. Furthermore, of the 2879 participants who were included in the final analyses, few participants reported a driving cessation event (n = 27). The true value of the hazard ratio comparing driving cessation among those who are frail to those who are not frail could vary greatly from the estimated hazard ratio of 6.08 (95% CI: 1.36–27.26). As discussed by Li et al. (2017), the composition of the AAA LongROAD cohort is not reflective of the overall U.S. population in terms of important demographic factors, such as race, socioeconomic status, and general well-being, and thus, findings may not be generalizable to the entire U.S. population [13]. Regarding potential measurement issues, while there may be arguments against the conversion of a continuous variable (annual mileage) to a dichotomous variable (low-mileage driver status), the interpretability of this study’s results was dependent upon this dichotomization. To date, all empirical research efforts on the low-mileage bias have evaluated the effect of being a low- or non-low-mileage driver on crash rates, rather than the effect of decreases in annual mileage viewed on a continuous scale [6,18,19].

In contrast to these limitations, this study offers important strengths. Due to the longitudinal nature of the data used in this study, the significant results indicate a potential causal relationship between baseline frailty status and low-mileage driver status along with time to driving cessation. Additionally, this study was bolstered by the availability of data on a variety of covariates that may have confounded the association of interest. 

Future studies should use crash records collected by the AAA LongROAD study to objectively evaluate the validity of these findings. Furthermore, future research conducted using data from the AAA LongROAD cohort will have access to additional longitudinal data, which will allow for a more thorough analysis of this relationship. Future interventions may include the potential ability to identify individuals who are at higher risk for low-mileage driving, and driving cessation has important quality of-life implications, since these adverse driving outcomes can potentially be mitigated, given the modifiable nature of the risk factors examined in this study. Public health interventions aimed at improving the modifiable components of the frailty phenotype may lead to mitigating crash rates and ameliorating driving cessation outcomes in older drivers.

## 5. Conclusions

The study supports the hypothesized association between poorer frailty status and reduced driving exposure. Analyses indicate that higher levels of frailty phenotype were associated with low-mileage driver status, and that those who are prefrail are at higher risk for driving cessation compared to those who are not frail. Interventions and programs that help reduce frailty phenotype among older adults may assist with reducing the likelihood of adverse driving outcomes.

## Figures and Tables

**Table 1:** Baseline characteristics of the American Automobile Association (AAA) LongROAD study population.

	N (%)
**Gender (N = 2964)**	
**Male**	1396 (47.10)
**Female**	1568 (52.90)
**Age (N = 2964)**	
**65–69**	1232 (41.57)
**70–74**	1027 (34.65)
**75–79**	705 (23.79)
**Marital Status (N = 2936)**	
**Married/Living with Partner**	1965 (66.93)
**Divorced/Separated/Never Married**	596 (20.30)
**Widowed**	375 (12.77)
**Education Level (N = 2955)**	
**High School or Less**	335 (11.34)
**Some College**	721 (24.40)
**Bachelor’s Degree**	690 (23.35)
**Advanced Degree**	1209 (40.91)
**Depression (N = 2960)**	
**≤55**	2774 (93.72)
**>55**	186 (6.28)
**Cognitive Health (N = 2883)**	
**0–10**	1493 (51.79)
**11–20**	1390 (48.21)
**Vision (N = 2962)**	
**Poor to Good**	973 (32.85)
**Very Good**	1249 (42.17)
**Excellent**	740 (24.98)
**Driving Importance (N = 2961)**	
**1 to 5**	109 (3.68)
**6**	404 (13.64)
**7–Completely**	2448 (82.67)
**Frailty Status (N = 2964)**	
**Frail**	86 (2.90)
**Prefrail**	1656 (55.87)
**Not Frail**	1222 (41.23)

**Table 2:** Associations between frailty status and low-mileage driver status.

**< 3000 miles per year**	
Frailty Status Frailty (not frail, prefrail, frail)	Adjusted Risk Ratio (95% CI)^1^ 1.36 (1.11, 1.65)
**< 1865 miles per year**	
Frailty Status Frailty (not frail, prefrail, frail)	Adjusted Risk Ratio (95% CI)^1^ 2.38 (1.63, 3.46)

^1^ Adjusted for age, gender, self-reported vision, cognitive health, and correlation within each site. N = 2706.

**Table 3:** Unadjusted and adjusted association of frailty status with time to driving cessation.

	Crude HR (95% Confidence Interval) ^1^	Adjusted Hazard Ratio (95% Confidence Interval) ^2^
Frailty Status		
Prefrail	4.04 (1.72–9.46)	4.15 (1.89–9.10)
Frail	8.25 (2.08–32.75)	6.08 (1.36–27.26)

^1^ Total N = 2879, Not Frail N = 1198, Prefrail N = 1601, Frail N = 80, and 1,557,710 person-days.

^2^ Adjusted for gender, self-reported vision, cognitive health, and driving importance and clustered by site. Total N = 2879, Not Frail N = 1198, Prefrail N = 1601, Frail N = 80, and 1,557,710 person-days.
